# Open questions on methane hydrate nucleation

**DOI:** 10.1038/s42004-021-00539-6

**Published:** 2021-07-01

**Authors:** Guang-Jun Guo, Zhengcai Zhang

**Affiliations:** 1 Key Laboratory of Petroleum Resource Research, Institute of Geology and Geophysics, Chinese Academy of Sciences, Beijing, P. R. China; 2 Innovation Academy for Earth Science, CAS, Beijing, P. R. China; 3 College of Earth and Planetary Sciences, University of Chinese Academy of Sciences, Beijing, P. R. China; 4 Laboratory for Marine Mineral Resources, Pilot National Laboratory for Marine Science and Technology, Qingdao, P. R. China

**Keywords:** Chemical physics, Natural gas

## Abstract

The commercial use of natural methane hydrate is hampered by several open questions that remain regarding hydrate formation. Here the authors comment on past interpretations and aim to provide a roadmap for developing a predictive theory of methane hydrate nucleation.

Methane gas contacting liquid water is a frequent natural scenario, e.g., when natural gases are released from seafloor sediments or when shale gas and coalbed gas permeates in water-bearing rock stratum. In chemical industries, water-bearing natural gases often flow through pipelines, or gas mixtures are separated by using water. Generally, methane gas can hardly dissolve in water because its solubility is as small as 10^−3^−10^−5^ mole fractions under the ambient conditions^1^. However, temperatures <10 °C combined with pressures >7 MPa are easily found on Earth. Then, methane molecules can dissolve in water with a ratio of 0.148 mole fractions, i.e., its solubility enhances over two orders of magnitude. The result is an ice-like crystalline phase termed methane hydrate, whose reservoirs are found in offshore sediments and permafrost, and that blocks oil and gas pipelines. The structure of methane hydrate is well known as sI^2^, that is, water molecules form two types of cages (5^12^, dodecahedron, and 5^12^6^2^, tetrakaidecahedron) with a ratio of 2:6 to stack the three-dimensional space like a mosaic. Methane molecules fill these cages one by one but with an uncertain occupancy. Intriguingly, the above reaction process of forming methane hydrate often requires an uncertain induction time, indicating a metastable state before the methane–water system begins to nucleate. A so-called memory effect exists—the second time of hydrate formation reaction is much faster than its first time after the first result is decomposed. Considering the basic thermodynamic properties of methane hydrate are well measured^2^, another fundamental question is: how does methane hydrate form? To answer this question on a molecular level is very important also for practical applications, as many hydrate-based techniques, including flow assurance, gas storage, mixture gases purification, and seawater desalination, utilize the formation and dissociation of hydrates inevitably. Moreover, the more complicated exploitation of methane hydrate as a huge energy resource eagerly requires to understand the kinetic behaviors of methane hydrate in depth.

Methane hydrate formation can be divided into two stages: embryo nucleation and crystal growth. The former is more difficult to be experimentally investigated than the latter because nucleation means a new phase developing “from nothing” and it occurs at the nanoscale in both spatial and temporal dimensions. Thus, recent contributions to methane hydrate nucleation are mainly based on molecular simulations. In this Comment, the popular hypotheses and mechanisms of methane hydrate nucleation together with the involved simulation efforts are overviewed briefly. Then, several open questions are discussed to stimulate future studies.

## Past works

To describe the possible mechanism of methane hydrate nucleation, Sloan et al. proposed the labile cluster hypothesis in 1994, which emphasizes the agglomeration of labile clusters, i.e., unstable water cages filled with a methane molecule in each^3^. Referring to the classical nucleation theory (CNT), when the agglomeration of labile clusters exceeds a critical size, methane hydrate begins to grow spontaneously. However, Trout et al. believed the labile clusters tend to disintegrate rather than agglomerate and proposed the local structuring hypothesis^4^. They stated in 2002 that the gas molecules can arrange in a local ordered configuration by chance, and, when the number of gas molecules in the local structure exceeds the requirement of a critical nucleus, the surrounding water molecules will adjust their orientations to form the critical nucleus. Many researchers had also long noticed that hydrates form usually at the vapor–liquid interface and thus emphasized methane hydrate nucleation must utilize the particularly high concentration of methane molecules at the interface^5,6^. Therefore, methane concentration is a key factor to trigger hydrate nucleation. This point is easily understood because the methane concentration in the hydrate phase is at least 100 times higher than in methane aqueous solution, and the gas–water interface is a favorable location for preconcentrated methane–water mixture. In 2009, Guo et al. proposed in their cage adsorption hypothesis (CAH)^7^ that the methane aggregation is owing to a water cage adsorbing dissolved methane molecules and the adsorbed methane on the cage faces will subsequently develop new cages for adsorbing more distant methane molecules. The adsorption interaction plays a role in concentrating methane not only at the nucleation stage but also at the growth stage. More importantly, CAH can predict that the critical methane concentration is about 0.044 mole fractions above which the methane hydrate nucleation may start. An amorphous phase may occur as an intermediate before the crystalline phase is reached.

Also in 2009, Walsh et al.^8^ reported the first milestone simulation of methane hydrate nucleation, with their trajectories actually reaching the amorphous phase of methane hydrate according to the late analysis^9^. In 2010, Molinero et al.^10^ and Kusalik et al.^11^ observed the two-step mechanism of methane hydrate nucleation via molecular dynamics simulations and believed that the hydrate nucleation is similar to the Ostwald process, and it is necessary to form an amorphous phase followed by annealing to the crystalline phase. Especially, Molinero et al. proposed that the system first develops a blob, a local dense region of water-separated methane molecules, and then an amorphous nucleus is born in it. However, Walsh et al.^12^ and Zhang et al.^13^ subsequently found that methane hydrate can nucleate in multiple pathways—either forming amorphous nucleus as an intermediate indirectly or forming crystalline nucleus directly. In 2020, Li et al.^14^ identified ternary water-ring aggregations (TWRAs) as more fundamental structures than water cages for describing these multiple nucleation pathways uniformly, and the dehydration of hydration layers of methane molecules in the blob is a key step.

Recently, we further found that the critical gas concentration predicted by CAH is just a minimum requirement, not enough to trigger hydrate nucleation for other gas hydrates, such as CO_2_ hydrate. Only when the gas molecules become slow enough and reach the common critical self-diffusion coefficient, hydrate nucleation could begin^15^. Therefore, we do believe that hydrate nucleation is controlled both thermodynamically and dynamically. An entropy penalty appears: when guest molecules dissolve in water, the system favors to overcome the nucleation barrier of free energy, and the corresponding slowdown of guest mobility favors the system to find the pathway to nucleate.

### Open questions

**Fig. 1:**
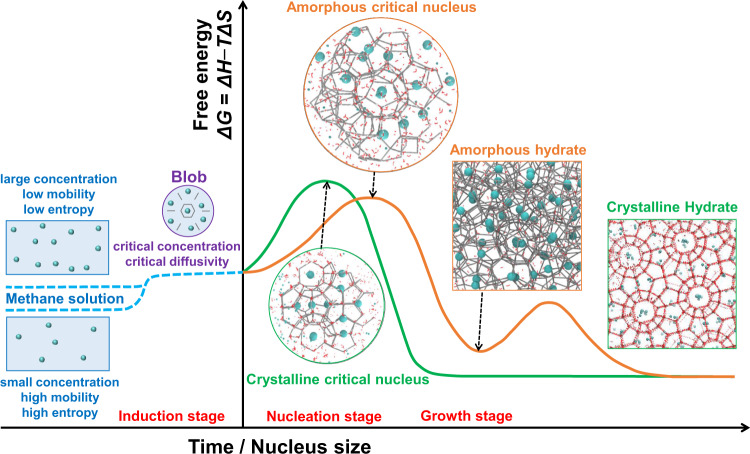
Schematic diagram showing methane hydrate nucleation. In the induction stage, increasing methane concentration will slow down molecular mobility, reduce system entropy, and increase free energy. When the critical concentration is reached in a local region near the gas–water interface, a blob forms with methane separated by water. At such high concentrations, water cages occur with a very large probability. Then, water cages adsorb surrounding methane molecules so as to concentrate the methane again. This process is spontaneous, and the adsorption interaction helps the blob to overcome energy barriers with forming a critical hydrate nucleus (i.e., cage clusters). Because the types of the first cage and the linking pattern of the subsequent cage clusters are uncertain, the critical nucleus’ structure is stochastic, leading to multiple pathways: directly forming the crystalline hydrate and indirectly forming the amorphous hydrate followed by an annealing/ripening process to ultimately reach the crystalline hydrate.

Based on the above overview, we summarize past findings on methane hydrate nucleation in Fig. 1. However, a theory is still unavailable to describe all sub-processes quantitatively. CNT cannot be applied to hydrate nucleation without any modification.

The first open question rests with the accurate description of the critical nucleus. Because the amorphous phase has various structures with different degrees of order (taking the crystallinity, occupancy, TWRAs, etc. as examples), the size of a critical nucleus may decrease with the nucleus ordering according to the predicted critical radius of CNT, i.e., *r*_c_ = −2*σ*/Δ*g*_v_, where *σ* is the surface free energy per unit area and Δ*g*_v_ is the free energy change per unit volume between the new phase and old phase. Therefore, structural ordering should be introduced into the calculations for both body free energy and surface free energy of a critical nucleus.

The second open question focuses on the nucleation stage. When the nascent cage in the blob begins to adsorb dissolved methane, the adsorption interaction will result in the blob releasing heat and reducing its entropy. It is a proper opportunity to predict the free energy barrier for methane hydrate nucleation and the evolutionary directions of the nucleus.

The third open question lies in the metastable methane solution in which the blob’s appearance may be a rate-controlling step with an induction time of 200–1200 ns, compared to the short nucleation stage of typical 10–100 ns^8,13^. How the local fluctuations of methane concentration make the blob appear may be a tough task to resolve.

Finally, the structural transition process from amorphous hydrate to crystalline hydrate also deserves to be studied, but we rank it at last because it is beyond the nucleation range.

### Outlook

In the future, it is deemed promising to develop the methane hydrate nucleation theory (MHNT) on the basis of CNT accompanied with answers to the above open questions. Along the roadmap (Fig. 1), ultimately the MHNT should be capable of predicting the critical concentration, critical diffusivity, critical nucleus, nucleation rate, and nucleation pathways quantitatively. Subsequently, the more general gas hydrate nucleation theory could be developed considering the particular features of different gas molecules, such as their size, structure, solubility, and diffusivity in water. Methane hydrate nucleation should be the best and most important benchmark to study other gas hydrate nucleation. Once our theoretical understanding of methane hydrate nucleation is mature, we expect gas hydrate use and applications to flourish tremendously.
